# Longitudinal associations between sport participation and fat mass with body posture in children: A 5-year follow-up from the Czech ELSPAC study

**DOI:** 10.1371/journal.pone.0266903

**Published:** 2022-04-11

**Authors:** Mario Kasović, Lovro Štefan, Pavel Piler, Martin Zvonar

**Affiliations:** 1 Department of General and Applied Kinesiology, Faculty of Kinesiology, University of Zagreb, Zagreb, Croatia; 2 Department of Sport Motorics and Methodology in Kinanthropology, Faculty of Sports Studies, Masaryk University, Brno, Czech Republic; 3 Department of Research and Examination (RECETOX), Faculty of Science, Masaryk University, Brno, Czech Republic; Universidad Pablo de Olavide, SPAIN

## Abstract

The main purpose of the study was to examine longitudinal associations between sport participation and fat mass with body posture in children. We used data from children recruited in the Czech European Longitudinal Study of Pregnancy and Childhood (CELSPAC) at the ages of 11 y (*n* = 1065), 13 y (*n* = 811) and 15 y (*n* = 974). Information on body posture, practicing sport in a club and at a competitive level, and skinfold thicknesses (biceps, triceps, subscapula, suprailiaca and thigh) from pediatrician’s medical records were collected. Body posture was inspected by a pediatrician. The sum of 5 skinfolds was used as a proxy of fat mass. The 85^th^ and 95^th^ percentiles defined ‘overfat’ and ‘obese’children. Practicing sport in a club and at a competitive level were included as ‘yes/no’ answers. General linear mixed models with risk ratios (RR) and 95% confidence intervals (95% CI) were calculated. Overall, 35.6% of children and adolescents had impaired body posture; the prevalence of ’incorrect’ body posture increased by age (from 41.0% to 28.0%, *p*<0.001). Practicing sport in a club and at a competitive level decreased by follow-up (*p*<0.001), while the level of ‘overfat’ and ‘obese’ children increased (*p*<0.01). In separate models, ’incorrect’ body posture was associated with non-practicing sport in clubs (RR = 1.68; 95% CI 1.43–1.97, *p*<0.001) or at competitive level (RR = 1.61; 95% CI 1.37–1.88, *p*<0.001) and with being ’overfat’ (RR = 2.05; 95% CI 1.52–2.75, *p*<0.001) and ’obese’ (RR = 2.15; 95% CI 1.68–2.75, *p*<0.001). When all variables were put simultaneously into the model additionally adjusted for sex, self-rated health and baseline body posture, similar associations remained. This study shows, that not participating in sport and being overfat/obese are longitudinally associated with ‘incorrect’ body posture. Therefore, the detection of these risk factors in childhood, through the development of school- and community-based interventions, should be advocated.

## Introduction

Postural problems have become one of the most common, yet still underestimated health problems globally [1]. Most postural problems refer to incorrect body posture in which the body cannot maintain a normal function of tissues and organs in an upright state [2]. Such problems often start to occur in childhood [3], and previous studies have shown that poor body posture tracks well from childhood to adulthood [4]. Evidence points out that 30% to 50% children and adolescents have different degrees of incorrect posture [5, 6], where the most prevalent posture problems include shoulder asymmetry, thoracic kyphosis and scapula tilt [1]. Untreated incorrect posture in childhood may be associated with the reduction in cardiorespiratory efficiency, decreased vital capacity of the lungs, degenerative bone and low back pains later in life [7].

The prevalence of overweight and obesity has dramatically risen in both developed [8] and less developed countries [9]. Estimates suggest that between 20% and 45% of European children are overweight or obese, with the highest proportion being observed in southern and central European countries [8]. In Czech Republic, it has been shown that the prevalence of overweight and obesity appears to rapidly increase, being between 20% and 30% in 11- to 15-year-old children and adolescents [10, 11]. Previous evidence shows, that being overweight or obese in childhood leads to health-related consequences later in life, including premature mortality [12]. Along with overweight and obesity, physical inactivity is considered to be one of the most important public health concerns globally [13]. Studies among children have consistently documented a steady decline in physical activity [14], especially between primary and secondary school [15]. Lower levels of physical activity also relate to adverse health-related outcomes in adulthood [16].

Overweight and obesity [1, 7–19] and low level of physical activity [3, 8, 20] have been associated with poor body posture in children. A study by Maciałczyk-Paprocka *et al*. [1] showed that 74% children with excessive body mass had postural errors more frequently, compared to children with standard body mass, where the most prevalent error locations included shoulders, abdomen, and head. Similar findings were obtained in another study, in which children with excessive body fat had less slope of the thoracic-lumbar spine, greater difference in the depth of the inferior angles of the scapula, and greater angle of the shoulder line. On the other hand, low physical activity might lead to a larger angle of body inclination [18]. In general, studies have shown that overweight and obese children with low physical activity engagement may experience a decrease in body stability, which can cause changes in habitual balance axis resulting in increased lumbar lordosis of abdominal protrusion and pelvic anteversion [18]. Unfortunately, the majority of studies exploring the associations between overweight and obesity and physical activity with body posture have been cross-sectional, and the longitudinal associations between the aforementioned health-related parameters are scarce.

The increasing epidemics of overweight and obesity and irregular physical activity, as well as the growing prevalence of incorrect body posture in children has resulted in calling for some actions, which would increase the level of physical activity and draw attention to embrace adequate dietary habits [1]. These modified activities may prevent from osteoporosis, osteoarthritis and back pain and other organ complications related to obesity [21]. Since excessive fat, irregular physical activity and poor body posture are common in children and track well to adulthood, it is necessary to establish time-to-time associations. By using a longitudinal design, the findings of this study would answer, whether body fat and irregular physical activity are associated with poor body posture from childhood to adolescence. If significant, health and school professionals might be able to implement special strategies and policies to target a ‘risky’ group of children by including them in physical education and extracurricular programs to decrease body fat, increase physical activity levels and improve body posture.

Therefore, the main purpose of the study was to examine longitudinal associations between, sport participation and fat mass with body posture. We hypothesized that overweight and obese children, who did not participate in sport would have worse body posture, compared to their normal weight peers who engaged in sport.

## Material and methods

### Study participants

Study data were derived from the Czech part of the ELSPAC study, a prospective birth cohort study carried out in the Czech Republic. The ELSPAC study investigated the effects of biological, psychosocial, economic and environmental factors on pregnancy, delivery and subsequent child’s development and health. The Czech part was approved for adherence to ethical guidelines by the Scientific Council of Masaryk University. The Czech cohort targeted the entire population of births in Brno and Znojmo regions of Czechoslovakia (now Czech Republic) between March 1, 1991, and June 30, 1992. The participation rate of invited pregnant women was 96%. All participating women provided written informed consent. The mother-child pairs were followed mainly by health records, examinations, and self—reported questionnaires completed by parents and later on by the children themselves. A total of 4811 mothers completed first self-reported postnatal questionnaires. Additional details about the study have been published earlier [22]. We based our data on pediatrician’s medical assessments at the ages of 11 y (*n* = 1065), 13 y (*n* = 811) and 15 y (*n* = 974). Pediatrician’s medical records included body posture inspection, sport participation in school, sport participation at competitive level, sum of 5 skinfolds, sex and self-rated health. The procedures performed in this study were anonymous and according to Declaration of Helsinki, also approved by the Ethical Committee of the RECETOX department of the Faculty of Science.

### Body posture

The assessment of prevalence of postural problems was performed according to the pediatrician’s body inspection at each systematic examination. The inspection was comprised of body posture characteristics, examined according to the visual assessment method, including appropriate functional tests to check the muscular constrictions. Each child was instructed to be in underwear and was analyzed in three planes (sagittal, frontal and transversal). The pediatrician assessed body posture from head to lower limbs; the alignment of head, neck, shoulders, spine curvatures (the course of lordosis and kyphosis), chest, abdomen, pelvis, thighs and calves. Similar to previous studies, the child standing with his/her back to the examiner was told to loosely bend forward (feet slightly apart, head and arms hanging down loose), after which the pediatrician reported if the child had scoliotic posture or not [1]. If no deviations were observed, the child was grouped into ‘correct’ *vs*. those who had at least one deviation as ‘incorrect’ body posture.

### Sport participation assessment

Practicing sport in a club and at a competitive level were assessed with two yes/no questions inquiring about regular participation in organized sport in sport clubs, as well as being engaged in sports at competitive level.

### Fat mass assessment

Skinfold thicknesses were measured using John Bull skinfold caliper (in mm) using a standardized procedure. We measured biceps, triceps, subscapula, suprailiaca and thigh skinfold thicknesses. For biceps skinfolds, the landmark was located at the level of the mid-point between the lateral edge of the acromion process and the radiale (elbow joint), on the mid-line of the front surface of the arm. The arm was relaxed with the palm of the hand facing forward. To measure triceps skinfolds, the landmark was located at the level of the mid-point between the lateral edge of the acromion process and the radiale (elbow joint), on the mid-line of the back surface of the arm. The arm was relaxed with the palm of the hand facing forward. Subscapula skinfold was measured by placing the landmark at the lower angle of the scapula, and the natural fold of the skin was pinched at about 45 degrees. The skinfolds of the suprailiaca was assessed by placing the landmark at the intersection between the spinale and the horizontal line at the level of the iliac crest. Finally, to measure thigh skinfolds, the landmark was placed at the midpoint of the surface of the thigh, between the patella and the crease at the top of the thigh. For the purpose of this study, the sum of 5 skinfolds was derived as the sum of biceps, triceps, subscapula, suprailiaca and thigh skinfold thicknesses, which has been used in previous studies [23]. We divided children for each year in three groups, according to the sum of 5 skinfolds as follows: (i) normal fat (<85^th^ percentile), (ii) overfat (≥85^th^—<95^th^ percentile) and (iii) obese (≥95^th^ percentile) [24]. The category ‘underfat’ was not presented, since only 2% of the participants were collapsed in this category and were therefore considered as ‘normal’ fat.

### Covariates

To assess health, the pediatrician asked each child about his/her general health:”How would you rate your health” with 5 possible answers: (i) chronically ill (refers to chronic illness diagnosed by a health professional), (ii) almost always ill, (iii) sometimes ill, (iv) healthy, with a few minor problems and (v) perfectly healthy. The outcome was dichotomized into ‘good’ (healthy, with a few minor problems and perfectly healthy) *vs*. ‘poor’ (chronically ill, almost always ill and sometimes ill) collected at the age of 11 y, 13 y and 15 y. Since baseline body posture might be affecting body posture measured at follow-up [4], the 2^nd^ model was adjusted for body posture inspected at the age of 11 y. Sex was coded as boys *vs*. girls.

### Data analysis

Before the data processing, we used the data cleaning and selection approach. At first step, we converted missing code to missing value, after which the data set was reshaped from long to wide format. By having the data converted in wide form resolves the nesting issue and enables to use variables from the other time periods as predictors of missing values. At the final stage, the data imputation was performed [25]. Basic descriptive statistics are presented as percentages (%). Categorical differences at the ages of 11 y, 13 y and 15 y were calculated using Chi-square test. To examine longitudinal associations between sport participation in sport clubs, at competitive level and sum of 5 skinfolds with ‘incorrect’ body posture, we used linear mixed models with time as a fixed effect. Linear mixed models account for the correlation between repeated measures of the same participants over time. Regression estimates (β coefficient) with 95% confidence intervals (95% CI) were estimated in all models using 500 cluster bootstrap samples to account for the dependence between repeated measures. A first order auto-regressive correlation structure was used to account for repeated measures. In addition, we calculated risk ratios (RR), to present the probability of having ‘incorrect’ body posture for all study variables. In unadjusted model (model 1), all study variables were put separately into the calculation. In model 2, practicing sport in a club and at a competitive level and sum of 5 skinfolds were put simultaneously into the analysis, additionally adjusted for sex and self-rated health, which were collected at each time point, and baseline body posture. The interaction terms between sex and body posture, practicing sport in a club, practicing sport at a competitive level and the sum of 5 skinfolds were not statistically significant (*p*>0.05) so we dropped the sex-stratified analyses. Two-sided p-values were used, and significance was set at α<0.05. All the analyses were calculated in Statistical Packages for Social Sciences v.23 (SPSS, Chicago, IL, United States).

## Results

Basic descriptive statistics are presented in Table 1. Similar proportion of boys and girls at the ages of 11 y, 13 y and 15 y was represented. At the baseline, more than half of the children was classified as having ‘incorrect’ body posture, with a rising tendency till the age of 15 y (*p*<0.001). Around one-third of all participants practiced sport in a club and at a competitive level. Based on the sum of 5 skinfolds, approximately 20% of children were overweight/obese at the age of 11 y, with an increasing trend across the age groups (24.5% and 26.5% at the ages of 13 y and 15 y). Moreover, higher percentage of boys were classified as having ‘incorrect’ body posture, compared to girls (59.4% *vs*. 40.6%), while similar proportion of overweight/obesity in both sexes was observed.

**Table 1:** Descriptive statistics of study participants at the ages of 11 y, 13 y and 15 y.

Study variables	11 y (*N* = 1065)	13 y (*N* = 811)	15 y (*N* = 974)	*p*-value*
	%	%	%	
**Sex**				
Girls	50.1	50.1	50.1	
Boys	49.9	49.9	49.9	1.000
**Body posture**				
Correct	40.0	37.6	29.2	
Incorrect	60.0	62.4	70.8	< 0.001
**Practicing sport in a club**				
Yes	39.9	34.9	29.1	
No	60.1	65.1	70.9	< 0.001
**Practicing sport at a competitive level**				
Yes	33.7	39.5	34.9	
No	66.3	60.5	65.1	0.026
**Sum of 5 skinfolds**				
Normal fat (<85^th^ percentile)	78.7	75.4	73.5	
Overfat (85^th^-<95^th^ percentile)	13.4	14.6	16.0	
Obese (≥95^th^ percentile)	7.9	9.9	10.5	0.006
**Self-rated health**				
Good	91.4	90.5	88.7	
Poor	8.6	9.5	11.3	< 0.001

*denotes using Chi-square test.

Longitudinal associations between practicing sport in a club and at a competitive level and overweight and obesity with ‘incorrect’ body posture are presented in Table 2. In model 1, children who did not practice sport in clubs, did not compete at competitive level and were overweight and obese had 1.68-, 1.61-, 2.05- and 2.15-times higher risk of having ‘incorrect’ body posture. When all the study variables were put simultaneously into the model 2 and adjusted for sex, self-rated health and baseline body posture, ‘incorrect’ body posture remained associated with non-practicing sport in clubs (RR = 1.90, 95% CI 1.55–2.36, *p*<0.001), not competing in any sport at competitive level (RR = 2.40, 95% CI 1.91–3.00) and being overweight (RR = 1.52, 95% CI 1.1–2.05, *p* = 0.020) and obese (RR = 1.62, 95% CI 1.29–1.99, *p* = 0.007). Of note, boys were 2.57 more likely to have ‘incorrect’ body posture, compared to girls, and those individuals who reported having ‘poor’ health and were diagnosed with ‘incorrect’ body posture at baseline were 1.91 and 2.07 more likely to have ‘incorrect’ body posture at follow-up.

**Table 2:** Longitudinal linear mixed associations between participation in sport clubs, sport participation at competitive level and sum of 5 skinfolds with ‘incorrect’ body posture in the study participants.

Study variables	‘Incorrect’ body posture				
	Estimate	Std. Error	RR	95% CI	*p*-value
***Model 1*** ^***a***^					
**Practicing sport in a club**					
Yes	Ref.				
No	0.52	0.06	1.68	1.43–1.97	<0.001
**Practicing sport at a competitive level**					
Yes	Ref.				
No	0.47	0.08	1.61	1.37–1.88	<0.001
** Sum of 5 skinfolds**					
Normal fat	Ref.				
Overfat	0.72	0.15	2.05	1.52–2.75	<0.001
Obese	0.77	0.13	2.15	1.68–2.75	<0.001
** Sex**					
Girls	Ref.				
Boys	0.83	0.09	2.30	1.93–2.75	<0.001
** Self-rated health**					
Good	Ref.				
Poor	0.66	0.17	1.94	1.39–2.70	<0.001
** Baseline body posture**					
Correct	Ref.				
Incorrect	1.39	0.19	4.02	2.76–5.85	<0.001
***Model 2*** ^***b***^					
**Practicing sport in a club**					
Yes	Ref.				
No	0.64	0.11	1.90	1.55–2.36	<0.001
**Practicing sport at a competitive level**					
Yes	Ref.				
No	0.87	0.11	2.40	1.91–3.00	<0.001
** Sum of 5 skinfolds**					
Normal fat	Ref.				
Overfat	0.42	0.16	1.52	1.12–2.05	0.020
Obese	0.48	0.20	1.62	1.29–1.99	0.007
** Sex**					
Girls	Ref.				
Boys	0.94	0.10	2.57	2.10–3.14	<0.001
** Self-rated health**					
Good	Ref.				
Poor	0.65	0.20	1.91	1.28–2.84	0.002
** Baseline body posture**					
Correct	Ref.				
Incorrect	0.99	0.15	2.07	1.50–2.68	<0.001

^**a**^**Model 1:** examines longitudinal associations between practicing sport in a club and at a competitive level and sum of 5 skinfolds, sex, self-rated health and baseline body posture entered separately into the analysis.

^**b**^**Model 2:** examines longitudinal associations between practicing sport in a club and at a competitive level and sum of 5 skinfolds, adjusted for sex, self-rated health and baseline body posture entered simultaneously into the model.

*p* ≤ 0.05 level of significance.

## Discussion

The main purpose of the study was to examine longitudinal associations between practicing sport in a club and at a competitive level and fat mass with body posture. Our main findings are: (i) not participating in sport clubs or not doing sport at competitive level and being overweight and obese are associated with ‘incorrect’ body posture; (ii) when all the study variables are put simultaneously into the model and adjusted for several potential covariates, similar associations remain.

Lower level of physical activity is often assumed to be responsible for the excessive accumulation of body fat, therefore these two adverse health factors often co-exist. Our study showed, that not participating in sport clubs and not doing sport at competitive level were associated with ‘incorrect’ body posture, which is in line with previous evidence [18]. It has been demonstrated, that children with lower level of physical activity may be suffering from more postural problems, a more ‘incorrect’ value of lumbar lordosis, the larger angle of the sacrum and smaller spinal range of motion in the sagittal and frontal planes, compared to children with higher level of physical activity [18]. Indeed, lower level of physical activity may be a predisposing factor for spinal deformity [20], leading to the reduction in motor skills and more disturbances in body posture [26] Previous evidence suggests, that regular physical activity program may improve children’s shape of the body, stability and vestibular system, which reduce the likelihood of falls [27].

Our findings also showed that children with overweight and obesity had higher risk for ‘incorrect’ body posture over a follow-up, compared to their normal weight peers. Recently, a study by Maciałczyk-Paprocka *et al*. [1] showed that 74% of children with excessive body weight had postural errors. For example, the majority of participants aged 13–18 years had serious orthopedic complications, including valgus knees and flat feet, which postural assessment may be useful in differentiating between physiological and pathological alternations [1]. Flat foot is a common postural problem in children with overweight and obesity, since their physical activity and fitness levels are lower and, along with overweight and obesity affect midfoot overload during gait [28]. On the other hand, a study by Rusek *et al*. [29] showed that children with higher body-mass index values were found to have a decreased distance between the scapula and the frontal plane. Higher prevalence of postural problems and overweight and obesity at the age of 15 y can be explained by the speed of puberty [1]. Between the ages of 13 y and 18 y, a rapid increase in body height and accumulated fat is observed. This has been confirmed by previous studies, where older children aged 13–15 years are reported to have asymmetry in the position of shoulders, which is associated with varied overloads and carrying schoolbags on one side, which is typically observed in adolescents [30].

The current study enabled the assessment of body posture in association to sport participation patterns and overweight and obesity. The period of school education marks very sensitive changes, due to the new co-occurring environmental factors [29]. The accumulated fat and lower level of physical activity are often accompanied by the long periods of seated position of the child in class and during other school-related activities. Moreover, the adverse risk-factors also include carrying heavy schoolbags with different loads, asymmetry of schoolbag straps and the position of a schoolbag worn by the child. Such behavioral patterns may promote different postural habits, including more pronounced thoracic kyphosis and downward head positioning [31]. Thus, impaired body posture can be a limiting factor for engaging in regular physical activity and spending more time in front of a computer or television. Since studies among school-going youth have consistently documented a steady decline in physical activity and an increase in sedentary behaviors throughout childhood [32], it is necessary to establish school-based interventions that promote organized sport participation (in school, outside of school) and weight management programs for less active children and those with overweight and obese in both primary and secondary school.

This study has several strengths. The findings presented in this paper may be used for comparative studies. Central European population of children born in the early 1990s grew up under specific socioeconomic and cultural conditions, which were very different from those in Western part of Europe [33]. A longitudinal design allows us to establish a certain causality between several time-points, and to observe changes in a given variable.

However, this study is not without limitations. First, more objective methods in assessing body posture should be used in future research. Second, maturity of children were not assessed at the baseline. Third, although body fat is often calculated from regression equations, previous evidence has shown a significant measurement error, when compared with the 4-component model [34]. Fourth, the models in the study were not adjusted for environmental factors, including objectively measured physical activity, dietary patterns and sedentary behaviors. Fourth, health was self-reported, and we cannot exclude the possibility of measurement errors. Finally, school-based behavioral patterns, like the type of schoolbag and its load were not measured.

In conclusion, not-participating in sport clubs, not being engaged in sport at competitive level and being overweight and obese are all longitudinal significant correlates of ‘incorrect’ body posture in children. Therefore, implementing the call for prevention programs addressing higher sport participation rate and lower fat mass content may have beneficial effects on future body posture in school-aged children and adolescents.

## Supporting information

S1 Raw data (XLSX)
